# Segmental Bioelectrical Impedance Spectroscopy to Monitor Fluid Status in Heart Failure

**DOI:** 10.1038/s41598-020-60358-y

**Published:** 2020-02-27

**Authors:** Matthias Daniel Zink, Fabienne König, Sören Weyer, Klaus Willmes, Steffen Leonhardt, Nikolaus Marx, Andreas Napp

**Affiliations:** 1 Department of Cardiology, Angiology and Internal Intensive Care Medicine, University Hospital, RWTH Aachen University, Pauwelsstr. 30, 52074 Aachen, Germany; 2 Chair for Medical Information Technology, RWTH Aachen University, Aachen, Germany; 3 Department of Neurology, University Hospital, RWTH Aachen University, Aachen, Germany

**Keywords:** Heart failure, Oedema

## Abstract

Bioelectrical impedance spectroscopy (BIS) measures body composition, including fluid status. Acute decompensated heart failure (ADHF) is associated with fluid overload in different body compartments. This investigation aimed to evaluate the feasibility of measuring and monitoring fluid accumulation in patients with ADHF using BIS. The extracellular impedance as a surrogate marker for fluid accumulation was measured in 67 participants (25 healthy reference volunteers and 42 patients admitted with ADHF) using BIS in the “transthoracic”, “foot-to-foot”, “whole-body” and “hand-to-hand” segments. At baseline, BIS showed significantly lower extracellular resistance values for the “whole-body” (*P* < 0.001), “foot-to-foot” (*P* = 0.03), “hand-to-hand” (*P* < 0.001) and “transthoracic” (*P* = 0.014) segments in patients with ADHF than the reference cohort, revealing a specific pattern for peripheral, central and general fluid accumulation. The “foot-to-foot” (AUC = 0.8, *P* < 0.001) and “hand-to-hand” (AUC = 0.74, *P* = 0.04) segments indicated compartments of fluid accumulation with good prediction. During cardiac recompensation, BIS values changed significantly and were in line with routine parameters for monitoring ADHF. Mean bodyweight change per day correlated moderately to good with BIS values in the “whole-body” (*r* = −0.4), “foot-to-foot” (*r* = −0.8) and “transthoracic” (*r* = −0.4) segments. Based on our analysis, we conclude that measuring and monitoring fluid accumulation in ADHF using segmental BIS is feasible and correlates with clinical parameters during recompensation.

## Introduction

Acute decompensated heart failure (ADHF) is typically associated with forward or backward pumping failure^1^. It is considered a severe health deterioration that frequently leads to hospital admission. Typical clinical symptoms of worsening heart failure are associated with central, peripheral or general fluid overload and include breathlessness and oedema. In addition to the treatment of the underlying cause of ADHF, removal of the excess fluid load in the body is a central aspect of ADHF management^1,2^.

However, individual clinical signs of volume overload, such as breathlessness and peripheral oedema, show wide variation. Thus, clinical presentation may be unspecific, but identifying fluid overload and ADHF^3^ is paramount to guide adequate treatment. To diagnose and monitor ADHF, the combination of biomarkers such as NT-proBNP^4^, bodyweight, ECG^5,6^, echocardiography^7,8^, functional^9^ and imaging^7,10^ findings in addition to the clinical aspect of the patient are considered pillars of clinical management. However, the identification and monitoring of fluid overload by these techniques are limited and mostly hampered by an overlap with concomitant critical health conditions^11^. Thus, differing impedance measurement techniques to determine body composition, fluid load and organ functionality by invasive^12–15^ or non-invasive^16–27^ approaches may be a potential supplement and extent to current diagnostic possibilities.

The electrical impedance of tissue is related to its fluid load and can be measured by an imperceptible current using bioelectrical impedance spectroscopy (BIS). In cases of high fluid load, as observed in cardiac decompensation, the impedance decreases. However, research on electrical impedance measurements focused mainly on one measuring segment such as the intrathoracic segment by invasive measurements or a thoracic or foot-to-hand segment by non-invasive measurements^23^. Unfortunately, fluid accumulation in compartments not appropriately covered by the measuring segment may, therefore, be neglected or, as body impedance is highly sensitive to body posture^25,28^, misinterpreted.

This feasibility study aimed to compare BIS values with the clinical diagnosis of fluid accumulation at admission and monitor fluid status during cardiac recompensation in multiple segments.

## Methods

### Ethical considerations

The study was carried out at the Department of Cardiology, Angiology and Intensive Care Medicine, University Hospital RWTH Aachen. The trial was approved by the institutional review board of the University Hospital RWTH Aachen (patient cohort: EK202/11; NCT 01775306; reference cohort: EK206/11; NCT 01778270). The investigation met the ethical principles based on the Declaration of Helsinki, current legal requirements (German medical devices act and code of medical ethics) and Good Clinical Practice guidelines. Informed consent was obtained from all participants.

### Description of participants, treatment, cohorts and study design

Initially, 44 consecutive eligible patients suspected of having ADHF with clinical signs for acute decompensation and at least 18 years of age were included. The exclusion criteria were pregnancy or lactation, implanted electrical devices or patients unable to provide consent. As a reference group, 25 healthy subjects at a single time point were measured.

Two patients had to be excluded because initial symptoms were not heart failure related. Full baseline measurements were obtained in 67 participants (42 patients and 25 healthy subjects). For clinical and logistic reasons, several repeated measurements varying in number were available for 36 patients. In this cohort, the authors performed 161 measurements, with a mean of 4.5 ± 1.9 per patient (Table 1). The full description of employed tools can be found in the Supplemental Material (Supplemental Material, chapter 1). Individual patient treatment followed recommendations of the latest ESC heart failure guidelines^1^ and was at the discretion of the treating physician. There were no restrictions on diagnostic or therapeutic options for participating patients. At day of admission, 75% received treatment with loop diuretics, 81% received either angiotensin-converting enzyme inhibitors or angiotensin-receptor inhibitors, and 78% received beta blockers. Cardiac glycoside (digoxin or digitoxin) intake was observed in 25% of patients who suffered from atrial fibrillation. Considering the whole measurement period, 94% of all patients received loop diuretics at least once.

**Table 1:** Baseline characteristics.

	Patient cohort	Reference cohort	*P*
	Mean ± SD/Percentage(*N*)	Mean ± SD/Percentage(*N*)	
*N* =	42	25	
Age [years]	76 ± 13	26 ± 3	<0.001
Gender [male]	55%(23)	64%(16)	0.458
Height [cm]	169 ± 8	179 ± 8	<0.001
Bodyweight [kg]	82 ± 21	76 ± 18	0.209
BMI [kg/m^2^]	28.8 ± 6.6	23.5 ± 3.6	<0.001
Measurements per patient (*N* = 36)	4.5 ± 1.9		
Heart rate [Beats per minute]	80 ± 19		
Blood pressure systolic/diastolic [mmHg]	127 ± 25/69 ± 16		
NT-proBNP [pg /ml]	7217 ± 10814		
LVEF [%]*	40 ± 13		
Fluid accumulation [peripheral]	29%(12)		
[central]	40%(17)		
[general]	31%(13)		
NYHA [II]	24%(10)		
[III]	50%(21)		
[IV]	26%(11)		

^*^Data of 40 echocardiography studies.

Measurements were performed by trained medical staff and engineers following a structured protocol (Supplemental Material, chapter 2). Every measurement was complemented by a short medical history and registration of clinical parameters. The measurements of each patient were performed at baseline and every 2–4 days ranging from a total of 2 to 10 measurements per patient with up to 15 days of hospitalization (Supplemental Material, chapter 3, Fig. S1). To remove slight variations due to respiration, 10 impedance measurements were taken per visit, and the result was averaged.

**Figure 1:**
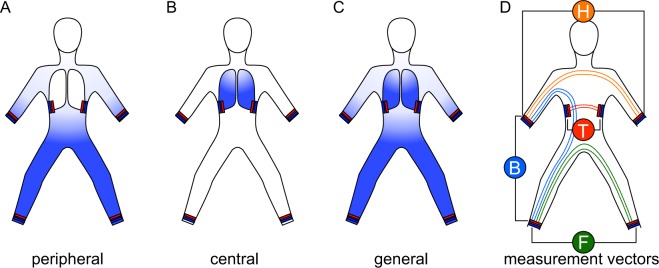
Fluid distribution and segments. At baseline, the cohort was separated into 3 groups related to the fluid overload identified by admission anamnesis and diagnostics (blue indicates fluid overload). (**A**) Peripheral oedema. (**B**) Central oedema. (**C**) Peripheral and central oedema considered as general fluid overload. (**D**) Each BIS measurement was performed in 4 segments: “foot-to-foot” (F, green), “hand-to-hand” (H, orange), “foot-to-hand” as a surrogate for the “whole-body” (B, blue) and “transthoracic” (T, red) segment (detailed description of electrode position: Supplemental Material, chapter 2).

To align the data with the clinical course, three time points were defined. Time point T1 was the baseline measurement, and time point T3 was the final measurement. The measurements between were averaged and considered as time point T2 (Supplemental Material, chapter 3, Fig. S1). At baseline, the patient cohort was divided into 3 groups related to the accumulation of fluid (Fig. 1A–C), and BIS values in 4 measured segments are shown in Fig. 1D.

- Peripheral oedema was defined by an anamnestic and qualitative increase in leg circumference with a test for a “pitting” oedema. Only oedema with persisting indentation was considered peripheral oedema.
- Central: Patients with central accumulation of fluid including both pulmonary congestion and pleural effusion determined at admission X-ray (Supplemental Material, chapter 4).
- General: Patients with signs for peripheral and central accumulation of fluid.

For patients with repeated measurements during clinical stay, the cohort was divided into two groups related to their outcome at the end of the hospital stay.

- Recompensated: For patients in whom initial signs of ADHF resolved and who were discharged from hospital care in a clinically stable and recompensated condition.
- Not recompensated: All patients who were not discharged at home after 15 days maximum. These patients showed either no clinical improvement or even further decompensation with the need for treatment at an intensive care unit within or at the end of the measurement interval.

### Bioelectrical impedance spectroscopy

Impedance measurement, as a non-invasive method to estimate body composition and fluid load, was developed in the second half of the 20^th^ century^29^. BIS is an established way to determine body composition^30,31^ – body fat in particular - in nutritional medicine^32,33^. With further development of the impedance measurement technique itself, it became a focus of research as a surrogate of fluid load and shift of various physiological and pathological conditions, such as body cell mass^34^ and pleural effusion^17,35,36^, respectively. The BIS measurement technique is described in detail elsewhere (Supplemental Material, chapter 5)^17,37^.

For all segments, we calculated the low-frequency domain as extracellular impedance [Ohm] because it correlates well with the extracellular water, which is mainly mobilized during diuretic therapy. Intracellular impedance is more influenced by the resistive component of cell membranes; therefore, individual composition of fat, muscle cells and age may distort measurements^31^.

### Statistical analysis

Statistical analysis was performed with IBM SPSS Statistics Version 25 (IBM Corporation 1994, 2019). Data are expressed as the mean ± standard deviation (SD) unless indicated otherwise. For baseline comparison, a one-way ANOVA was calculated. The Mann-Whitney U test for independent samples or the Wilcoxon rank-sum test for dependent data was used for pairwise comparisons. To determine the correlation among BIS values, bodyweight and NT-proBNP, the individual average change per day was calculated, and Pearsons linear correlation coefficient *r* was employed. *P* < 0.05 was defined as statistically significant. For two patients, echocardiography was insufficient; therefore, these two measurements were discarded, and only the data from 40 echocardiography studies are presented.

## Results

**Figure 2:**
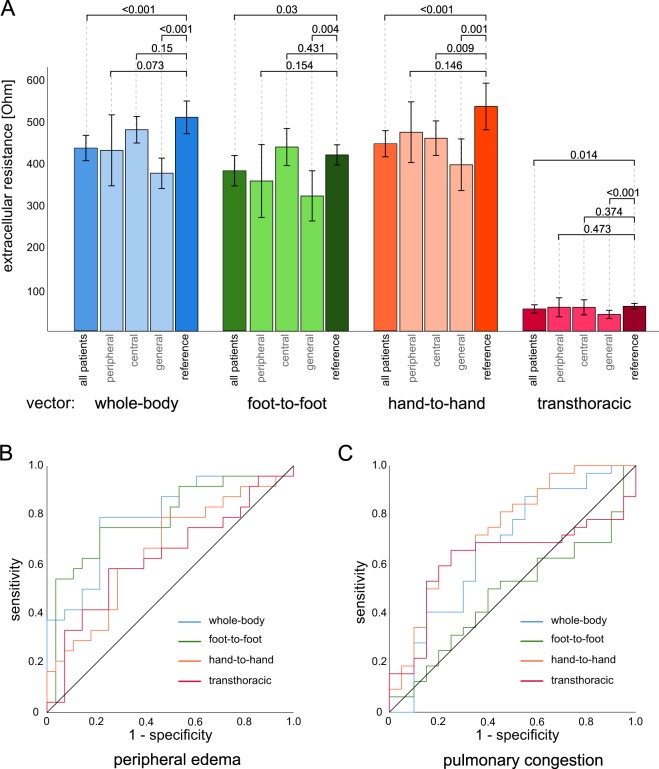
Segmental BIS measurements revealed specific patterns related to the fluid overload in body compartments. (**A**) For participants with clinical peripheral oedema (N = 12), baseline extracellular resistance was depressed in the “whole-body” and “foot-to-foot” segments. Patients with central oedema showed significantly lower extracellular resistance in the “hand-to-hand” (*P* = 0.009) segment. Patients with general fluid accumulation showed significantly lower extracellular resistance in all segments compared to the reference group. (**B**) Identification of peripheral fluid accumulation with good prediction for the “whole-body” (AUC = 0.8) and “foot-to-foot” (AUC = 0.79) segments. (**C**) Identification of pulmonary congestion as central accumulation with moderate to good prediction “whole-body” (AUC = 0.7, *P* = 0.044, S = 69%, F = 65%), “hand-to-hand” (AUC = 0.74) and “transthoracic” (AUC = 0.62) segments.

The baseline characteristics of all 67 participants are shown in Table 1. At baseline, extracellular impedance for all groups with fluid accumulation was significantly lower compared to the reference group (“whole-body” *P* < 0.001; “foot-to-foot” *P* = 0.03; “hand-to-hand” *P* < 0.001; “transthoracic” *P* = 0.014; Fig. 2A). Patients in the subgroup with general fluid accumulation showed the highest and most pronounced difference in all measured segments compared to the reference group (“whole-body” *P* < 0.001; “foot-to-foot” *P* = 0.004; “hand-to-hand” *P* = 0.001; “transthoracic” *P* < 0.001; Fig. 2A). Additionally, patients with central fluid accumulation showed significantly lower extracellular resistance in the “hand-to-hand” segment (*P* = 0.009) from the reference group. By comparing the impedance values of each measurement, there is a trend for patients with peripheral oedema to lower “whole-body” and “foot-to-foot” impedance values, whereas for central oedema, the impedance values in the “hand-to-hand” and “transthoracic” segments were lowered. Following the assumption of a general fluid overload, the impedance values of the group with both peripheral and central fluid accumulation were lower across all segments. At baseline, extracellular resistance for “foot-to-foot” (AUC = 0.79, *P* < 0.001, sensitivity *S* = 75%, specificity *F* = 79%, Fig. 2B) allowed us to distinguish between patients with and without peripheral oedema, whereas extracellular resistance for the “hand-to-hand” (AUC = 0.74, *P* = 0.004, *S* = 72%, *F* = 65%, Fig. 2C) distinguished patients with and without pulmonary congestion. Whole-body resistance indicated peripheral oedema (AUC = 0.8, *P* < 0.001, *S* = 79%, *F* = 79%, Fig. 2B) and pulmonary congestion (AUC = 0.7, *P* = 0.044, *S* = 69%, *F* = 65%, Fig. 2C) significantly.

### Monitoring clinical course

In 36 patients, repeated monitoring during in-hospital cardiac recompensation was possible. The monitoring group was retrospectively allocated into “recompensated” and “not recompensated” groups related to the clinical status at the end of the measuring period.

At baseline, the BIS values were not significantly different between the “recompensated” and the “not recompensated” groups. Furthermore, it was not possible to predict the recompensation outcome by the BIS baseline measurement.

For the “not recompensated” group, NT-proBNP levels decreased significantly from baseline to last measurement (*P* = 0.028, Table 2), and “transthoracic” impedance values reacted accordingly from T1 vs. T2 with a significant increase (*P* = 0.043, Table 2). This indicates a decreased fluid load for the thoracic compartment with less stretch on the cardiomyocytes, leading to a decreased release of NT-proBNP. Interestingly, all other BIS and clinical parameters showed no significant differences for the group with “not recompensated” clinical course at the end of the measurement period, including an unchanged amount of fluid in the remaining measured compartments.

**Table 2:** BIS and clinical parameters for all time points.

Time point	T1	T2	T3	T1 vs T2	T2 vs T3	T1 vs T3
	mean ± SD	mean ± SD	mean ± SD	*P*	*P*	*P*
	**Not recompensated**					
B (R_e_) [Ohm]	477 ± 115	469 ± 106	461 ± 108	0.866	0.735	0.508
F (R_e_) [Ohm]	405 ± 138	434 ± 115	398 ± 150	0.735	1	0.575
H (R_e_) [Ohm]	477 ± 111	484 ± 90	475 ± 98	0.612	0.499	0.959
T (R_e_) [Ohm]	59 ± 38	63 ± 50	57 ± 39	0.043	0.176	0.575
Bodyweight [kg]	87.4 ± 41.6	86.9 ± 43.7	87.4 ± 43.6	0.786	0.786	0.686
Heart rate [bpm]	80 ± 9	80 ± 15	82 ± 17	0.553	0.672	0.905
NT-proBNP [pg/ml]	8588 ± 10813	3991 ± 3478	2522 ± 2189	0.18	0.655	0.028
	**Recompensated**					
B (R_e_) [Ohm]	420 ± 94	468 ± 96	509 ± 113	0.006	0.001	<0.001
F (R_e_) [Ohm]	362 ± 111	418 ± 120	472 ± 128	0.001	0.002	<0.001
H (R_e_) [Ohm]	444 ± 102	491 ± 93	503 ± 124	0.007	0.072	0.009
T (R_e_) [Ohm]	45 ± 20	52 ± 17	58 ± 30	0.054	0.079	0.003
Bodyweight [kg]	79.4 ± 16.4	77.7 ± 16.6	76 ± 16.3	<0.001	0.001	<0.001
Heart rate [bpm]	80 ± 22	77 ± 15	72 ± 19	0.871	0.05	0.108
NT-proBNP [pg/ml]	6644 ± 11506	6940 ± 10531	4488 ± 6581	0.06	0.347	0.023

B – “whole-body” segment; F – “foot-to-foot” segment; H – “hand-to-hand” segment; T – “transthoracic” segment; R_e_ - extracellular impedance.

**Figure 3:**
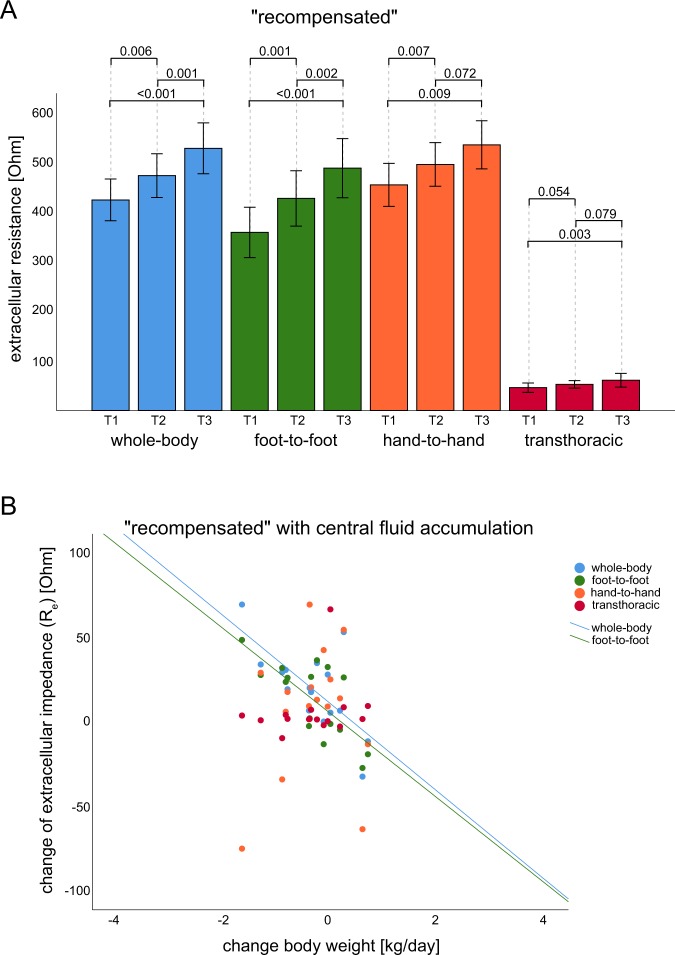
Timely change in BIS values in the measured segments of the “recompensated” group. (**A**) Course of BIS measurement for the “recompensated” group at all time points. All measured segments showed a significant increase from T1 to T3 as a result of diuretic therapy. (**B**) Mean change in bodyweight [kg] per day and mean change in BIS values for the “central” fluid accumulation subgroup showed a good linear correlation for the “whole-body” (r = −0.429, *P* = 0.036) and “foot-to-foot” (r = −0.787, *P* < 0.001) segment, while the “hand-to-hand” and “transthoracic” segment showed no significant correlation. For the significant correlation in the “whole-body” and “foot-to-foot” segment, the linear correlation line is shown. T1 baseline measurement, T2 average of all measurements between baseline and final measurement, T3 final measurement.

In the group with the “recompensated” clinical course, routine parameters for monitoring cardiac recompensation, such as bodyweight, heart rate, and NT-proBNP, improved significantly (Table 2). The BIS values changed in line with the routinely used parameters for all measured segments (Table 2, Fig. 3) for recompensated patients.

To determine the correlation between the changes in bodyweight, NT-proBNP and BIS measurements, mean changes per day were calculated for each subgroup of baseline fluid accumulation (“peripheral”, “central” and “general”). The mean daily change in BIS measurement correlated significantly with the change in bodyweight for the “whole- body” (subgroup “central” (*N* = 27): *r* = −0.429, *P* = 0.036), “foot-to-foot” (subgroup “central” (*N* = 27): *r* = −0.787, *P* < 0.001, Fig. 3B) and “transthoracic” (subgroup “peripheral” (*N* = 31): *r* = −0.374, *P* = 0.05). The BIS measurements showed no linear correlation with the mean change in NT-proBNP per day. Examples of individual clinical and impedance courses are presented in Supplemental Material chapter 6, revealing individual improvements in measured body compartments in line with clinical parameters.

## Discussion

Previous work showed promising results for the potential of invasive^12–15^ or non-invasive^18–23^ impedance measurements in heart failure patients at admission and in the prediction of recurrence. However, these studies were mainly focused on a single time point or single segment measurements. The purpose of this study was to test the feasibility of determining segmental fluid accumulation related to clinical findings at baseline and to analyse fluid loss and shift during recompensation for ADHF.

All patients were admitted with an initial diagnosis of ADHF, suffering from clinical signs of heart failure. The patient cohort was split according to the clinical examination at baseline into subgroups of dominant fluid accumulation labelled “peripheral”, “central” or “general”. On admission, BIS measurements were significantly lower for all measured segments compared to the healthy reference cohort. The analysis of subgroups revealed a specific pattern of affected segments of fluid overload: for the “peripheral” group “whole-body” and “foot-to-foot” BIS values; for the “central” group “hand-to-hand” and “transthoracic” values; and for the “general” group, all BIS values were lowered. The corresponding receiver operating curve analysis indicated that the “whole-body” (AUC = 0.8) and “foot-to-foot” (AUC = 0.79) segments were well able to predict “peripheral” oedema. The “whole-body“ (AUC = 0.7), “hand-to-hand“ (AUC = 0.74) and “transthoracic“ (AUC = 0.62) segments showed moderate to good prediction to assess “central” fluid accumulation as an important indicator for pulmonary congestion. The segmental BIS measurement allows additional insight of fluid accumulation compared to single segment measurement^12–15^ and performs well with clinical presentation of decompensation.

In a subgroup of 36 patients, repeated monitoring with 4.5 ± 1.9 BIS measurements on average were carried out comprising 161 measurements in total. Patients with repeated measurements were retrospectively allocated into a group of “recompensated” and “not recompensated” clinical courses as described in the methods section. Three measurement time points were considered (T1–T3). For the “not recompensated” group, no significant changes in bodyweight or BIS values were observed, except for a minor difference for the “transthoracic” segment between T1 and T2. In contrast, in the “recompensated” group, bodyweight and NT-proBNP levels decreased significantly from T1 to T3. In line with a clinical recompensation and routine parameters, BIS values significantly increased in all measured segments over all time points related to the loss of fluid with high sensitivity. BIS measurements could detect continuous significant changes and reflect the individual course of cardiac recompensation.

Compared to routinely used clinical parameters, such as bodyweight and NT-proBNP, the obtained impedance values were in line with these reference parameters for recompensated patients. The performance of NT-proBNP to detect and monitor heart failure remains questionable^23^; in our cohort, NT-proBNP values at baseline indicated heart failure and were in line with clinical diagnosis of acute cardiac decompensation. During in-hospital treatment, NT-proBNP values decreased in the “recompensated” and “not recompensated” (Table 2, Supplemental Fig. S3,A,B) groups but were not able to distinguish between the clinical outcomes in our cohort. A possible explanation could be that the reduction in central congestion leads to lower NT-proBNP values, which is reflected by a change in thoracic impedance in “recompensated” and “not recompensated” subgroups. However, in contrast to the “recompensated” subgroup, the fluid in the “not recompensated” group seems to shift in other body compartments, indicated by no substantial changes in “whole-body” values or bodyweight, indicating a persistent general fluid overload. It is known that NT-proBNP in the presence of impaired renal function can show extremely elevated NT-proBNP levels that do not necessarily match the degree of congestion^38,39^.

The insight in dynamic fluid load by BIS measurement is independent of the influence of factors such as kidney failure, which is, in our point of view, a major advantage and novelty. The segmental BIS measurement has the potential to overcome the limitations of single segment measurements^25^ and adds additional insights into fluid accumulation location. These findings should be verified in upcoming larger heart failure cohorts. Moreover, novel aspects of segmental BIS measurements reveal a specific pattern of fluid accumulation along with the clinical presentation on admission. Another important finding concerns the different time points of changes in BIS values. The “transthoracic” and “hand-to-hand” segments had a tendency to change immediately and increased significantly by the initiated treatment in individual courses (Supplemental Material, chapter 6), whereas the “whole-body” and “foot-to-foot” segments changed more slowly with a continuous linear increase, closely mirroring the course of bodyweight and NT-proBNP levels. Additionally, BIS measurements showed subgroup-specific linear correlations with the change in bodyweight per day (Fig. 3). An established gold standard in clinical routine for non-invasive fluid monitoring is not yet available and routinely used diagnostics such as bodyweight, and NT-proBNP provide only limited and coarse information regarding fluid accumulation. BIS measurements are investigator-independent, easy to record, harmless and not expensive, but should be performed under controlled circumstances to obtain robust data. In our investigation, BIS measurements revealed their full potential when employed as a repeated monitoring tool with segmental measurements, encouraging further investigations.

### Study limitations

This investigation should be considered as a feasibility study because of the small study cohort with diverse patient characteristics. The authors compared BIS measurements to routine clinical parameters and not to more sophisticated methods, such as total body potassium, isotope dilution or dual-energy X-ray absorptiometry, because they have specific limitations and are not established in clinical routine. Our reference cohort was not age-matched to our patient cohort, and the results may be influenced by differing distributions of muscle, fat, height, and weight. Our intention for the presented reference cohort was the lack of existing BIS reference parameters; therefore, we sought to show ideal values. Thus, the baseline comparison should not be seen in a confirmatory sense but as a potential application. Thus far, BIS does not provide a quantitative assessment of fluid volume overload. Until BIS is validated for quantitative fluid analysis, the technology will be limited to guiding in-hospital treatment, such as to what extent diuresis should be carried out. While possibly promising using the approach studied, this approach does not yet provide a diagnostic tool that can reliably support clinical decision making. Nevertheless, the authors followed a strict protocol with frequent individual measurements. Therefore, the authors consider the presented BIS results to be reasonable and robust.

## Conclusion

We conclude that in-hospital monitoring of cardiac recompensation using segmental BIS measurement in heart failure patients is feasible and shows correlation with the clinical course. Segmental BIS measurements provided data at baseline of fluid accumulation related to clinical finding of congestion. BIS measurement reacted during the recompensation process as routinely used parameters and provided additional information for location of fluid accumulation and shift within the body during treatment.

## Supplementary information

Supplemental material.

## Data Availability

The data generated for this study will be made available for further analysis upon reasonable request.
