# Supplementary material for: Segmental Bioelectrical Impedance Spectroscopy to Monitor Fluid Status in Heart Failure
Source: Sci Rep. 2020 Feb 27;10:3577. doi: 10.1038/s41598-020-60358-y (PMC7046702; doi:10.1038/s41598-020-60358-y)
Supplement: Supplementary file 1 — Supplemental material. [file 41598_2020_60358_MOESM1_ESM.docx]

Supplemental material

**Segmental Bioelectrical Impedance Spectroscopy to Monitor Fluid Status in Heart Failure**

*Matthias Daniel Zink¹*, MD; Fabienne König¹, MD; Sören Weyer^2^, PhD; Klaus Willmes^3^, MD; Steffen Leonhardt^2^, MD, PhD; Nikolaus Marx¹, MD; Andreas Napp^1^, MD*

*¹Department of Cardiology, Angiology and Internal Intensive Care Medicine, University Hospital, RWTH Aachen University, Pauwelsstr. 30; 52074 Aachen; Germany; ^2^Chair for Medical Information Technology, RWTH Aachen University, Germany; ^3^Department of Neurology, University Hospital, RWTH Aachen University, Germany*

1. **Full description of tools employed**

All measuring devices were arranged in a measuring cart (“IntelliVue MX800 Patient Monitor”, Koninklijke Philips N. V., Amsterdam. Netherlands) and data was captured with OpenClinica (Open Clinica. LLC. Waltham. MA. USA). Electrical integrity EN IEC 60601-1 was approved by the “Verband der Elektrotechnik, Elektronik und Informationstechnik” (VDE e.V., Frankfurt. Germany). Bioelectrical impedance measurements were performed with a commercially available spectrometer (SFB7; ImpediMed Ltd., Brisbane, Australia, CE certified) and BCM disposable electrodes “M35 143 1” (Fresenius Medical Care AG & Co. KGaA, 61346 Bad Homburg v.d.H., Germany).

1. **Measuring conditions**

In order to record reliable results, each measurement has to be recorded fulfilling the following conditions:

**Before measurement**

- Measurement takes place between 6-8 a.m.
- Before breakfast
- Patient rested at least 30 minutes in bed before measurement

**Preparation of measurement**

- Head of bed elevation is 30 degrees
- Arms are positioned sideways to the body and the back of the hand should point upwards
- Legs should be slightly spread and in straight position
- Arms and legs should have no skin contact to other parts of the body to avoid short circuits
- Position of the electrodes has to be unclothed and sanitized
- Electrodes have to be placed at the marked position of the previous measurement

**Electrode positions:**

**Hand electrodes:**

- The proximal electrode was placed at the wrist between Processus styloidei ulnae and radii, orthogonal to the length axis of the arm.
- The distal electrode was placed parallel to the proximal electrode on the back of the hand above the metacarpal bones with a distance of 5 cm.

**Foot electrodes:**

- The proximal electrode was placed at the height of the Malleolus medialis and lateralis.
- The distal electrode was placed parallel to the proximal electrode on the back of the foot above the metatarsal bones with a distance of 5 cm.

**Thoracic electrodes:**

The electrodes where place laterally on the chest above Musculus serratus anterior.

- The power fed electrode was placed in the 5^th^ intercostal space in the mid axillary line.
- The power deriving electrode was placed 5 cm ventral and parallel to the power fed electrode.

**During measurement**

- Patient should rest during measurement, avoid to speak and breath calmly
- Record of impedance measurement for 5 minutes

**After measurement**

- Mark the electrode position for the following measurement and remove the electrodes

1. **Time points of measurement**

At the day of admission because of acute decompensation or the day an in-house patient became symptomatic for acute decompensation of cardiac failure, we obtained informed consent. Despite the advantages of bioelectrical impedance spectroscopy (BIS), several factors can affect the measurements. Therefore, the measurements were performed in the morning before breakfast or the first turn out, to measure under laboratory conditions as constantly as possible.

The first and baseline measurement (Time point 1) was performed in the following time window at 6-8 a.m. This measurement was repeated every 2-4 days in the morning between 6-8 a.m. until discharge of the patient or complete reconvalescence of symptoms from cardiac failure during a longer lasting hospital stay. The data of measurements between baseline and discharge were averaged (Time point 2). The last measurement was considered as discharge measurement (Time point 3).

Figure S 1 Time points of measurement

1. **Diagnosis of central fluid accumulation:**

Chest X-Ray was performed in standing or upright position in two axes and diagnosed by a specialized radiologist. To diagnose pulmonary congestion criteria for pulmonary congestion were defined according to the textbook of Lange et al. (Lange S. Radiologische Diagnostik der Thoraxerkrankungen. 4th ed. Stuttgart: Thieme; 2010). Including, but not limited to:

- widened pulmonary artery
- increased vascularization
- "washed-out" Hilus
- marked interlobium
- basal haze
- elevated diaphragm
- cephalized perfusion
- bronchial collar
- Kerley A and B lines
- cardiac enlargement
- pleural effusion

1. **Bioelectrical impedance spectroscopy**

Biological tissues and organs have the property to conduct an administered current. Cells, membranes, free fluid, transition, and surface zones act like parallel and serially connected capacitors. The potential drop due to this combination of capacitors can be measured by the complex impedance (Z), which is a composite of ohmic resistance (R) and reactance (X). The magnitude of Z can be obtained from:

$$Z^{2}=R^{2}+X^{2}$$

Here, the resistance (R) acts as a resistor without capacitive features and is measured by the drop of input voltage. This is mainly influenced by fluids. In contrast, the reactance (X) behaves like a capacitor leading to a phase shift of the administered alternating current. Described as a circuit (see **Figure S2**), the possible pathways of biological tissues are predominantly influenced by the frequency of the administered current. The low frequencies preferentially use extracellular pathways, because they cannot overcome the high electrical resistance of the cell membranes (C_m_). As such, low frequencies represent the impedance of the extracellular water and represent the resistive component (R_e_). High-frequency current, on the other hand, moves straight through most biological tissues and allows calculation of intracellular impedance (R_i_).

Figure S 2 Pathways of electric current administered to biological tissue are mainly influenced by its frequency: Extracellular resistance (Re); Intracellular resistance (Ri); cell membrane (Cm)

## For bioelectrical impedance spectroscopy, we administered an alternating current of 200 µA in the frequency range of 5 kHz to 1000 kHz and measured the signal with a sampling frequency of 256/s. Resistance was then calculated using the Cole-Cole impedance model (0 to ∞ kHz parallel model).

The extra- and intracellular resistance can therefore be seen as an approximation for the impedance of the extra- and intracellular compartments according to fluid load. Cole provides a model to calculate the extra- and intracellular resistance for multiple frequencies.

$$Z(j\omega)=\frac{R_{e}}{R_{e}+R_{i}}\cdot(R_{i}+\frac{R_{e}}{1+{(\left( j\omega C_{m} \right)\cdot\left( R_{e}+R_{e} \right))}^{\alpha}})$$

With jω - imaginary frequency of the alternating electrical current

C_m_ –lumped cell membrane capacity

α - empiric factor capturing deviations from the simple R||RC circuit

In this study, non-linear least squares fitting has been used to minimize the summed squared of the error between the measured data point and the Cole model. The measured data in the frequency range were fitted to the non-linear parametric model of Cole, using the frequency ω as variable. Based on the Cole model, extracellular resistance (R_e_) is interpolated at very low frequencies (f = 0 kHz) with the assumption:

$$R_{0}=R_{e}$$

1. **Individual course of impedance measurements**

To visualize the feasibility of monitoring cardiac recompensation by different BIS segments, **Figure S3** displays relative changes to baseline for all BIS segments, body weight, and NT-proBNP simultaneously in two patients with 7 measurements each. As can be seen in **Figure S 3A** for a patient with central fluid accumulation and dyspnea as the leading initial symptom, the “transthoracic” segment showed an immediate response to the initiated treatment and the reduction of the fluid load within this segment and mirrored roughly the course of the NT-proBNP parameter. Loss of body weight was linear and was reflected by the “whole-body”, “foot-to-foot” and “hand-to-hand” segments mirroring this reduction of volume load.

For the patient with initial peripheral fluid accumulation (**Figure S 3B**), the “foot-to-foot” segment indicates best the reduction of fluid load. The other segments showed an increase related to the reduction in body weight and the decrease in NT-proBNP. During recompensation, the “transthoracic” segment showed a decrease and increase of BIS value related to the central fluid load.

**Figure S3** Individual patient’s course of relative changes of BIS measurement, body weight and NT-proBNP, schematic view of volume load to visualize the individual course for each time point (blue color indicates fluid accumulation). On the y-axis 1.0 indicates the value of initial measurement. **Figure S3A**: Patient with central fluid accumulation and dyspnea as leading initial symptom. BIS of “whole-body”, “hand-to-hand” and “foot-to-foot” were in line with the recompensation treatment with a steady increase in impedance over time, mirroring the continuous decrease in body weight. The “transthoracic” impedance measurement showed an immediate increase at measurement time point 2 while NT-proBNP decreases accordingly. At time point 3 NT-proBNP and “transthoracic” segment showed a kind of plateau phase and from time point 4 to 7 further increase of impedance and decrease of NT-proBNP level. **Figure S3B:** Patient with peripheral oedema as leading initial symptom. While the upper segments “hand-to-hand” and “transthoracic” showed an oscillation of the relative changes compared to baseline, “foot-to-foot” segment indicated best the effect of recompensation treatment with a steady increase of impedance. “Whole-body” impedance increased with a time-lag starting at time point 5 where body weight started to decrease. Since the upper-segments are not changing much, only small relative changes were observed for the NT-proBNP level.
